# Эктопическая акромегалия вследствие нейроэндокринных опухолей легких: первое описание в России трех клинических случаев

**DOI:** 10.14341/probl13346

**Published:** 2024-02-14

**Authors:** Е. О. Мамедова, Е. Г. Пржиялковская, С. А. Бурякина, Е. В. Бондаренко, А. М. Лапшина, М. Ю. Пикунов, Ж. Е. Белая, Г. А. Мельниченко

**Affiliations:** Национальный медицинский исследовательский центр эндокринологии; Национальный медицинский исследовательский центр эндокринологии; Национальный медицинский исследовательский центр эндокринологии; Национальный медицинский исследовательский центр эндокринологии; Национальный медицинский исследовательский центр эндокринологии; Национальный медицинский исследовательский центр хирургии имени А.В. Вишневского; Национальный медицинский исследовательский центр эндокринологии; Национальный медицинский исследовательский центр эндокринологии

**Keywords:** эктопическая акромегалия, нейроэндокринные опухоли, гормон роста-рилизинг гормон, первичный гиперпаратиреоз, макроаденома гипофиза

## Abstract

Акромегалия — нейроэндокринное заболевание, возникающее вследствие избыточной продукции гормона роста (соматотропного гормона, СТГ). В большинстве случаев причиной акромегалии являются аденомы гипофиза, продуцирующие СТГ. Гораздо реже встречаются случаи эктопической акромегалии. Последняя возникает вследствие опухолей, продуцирующих гормон роста-рилизинг гормон (ГРРГ), или опухолей внегипофизарной локализации, продуцирующих СТГ. Основными источниками секреции ГРРГ являются нейроэндокринные опухоли (НЭО) легких и поджелудочной железы. Лечение эктопической акромегалии заключается в хирургическом удалении источника гиперпродукции ГРРГ, а в случае невозможности радикального удаления используют аналоги соматостатина длительного действия, пэгвисомант, химиотерапию, иммунотерапию, лучевую терапию.

В данной статье приведено описание трех клинических случаев эктопической акромегалии вследствие ГРРГ-продуцирующих НЭО легких, каждый из которых примечателен рядом особенностей. В первых двух случаях акромегалия имела мягкое течение и неяркие клинические проявления, при этом во 2 случае имелось сочетание эктопической акромегалии и первичного гиперпаратиреоза. В 3 случае эктопическая акромегалия сочеталась с макроаденомой гипофиза, и после хирургического лечения НЭО ремиссия акромегалии не наступила. Во всех трех случаях НЭО легких были обнаружены случайно при проведении рентгенологического обследовании грудной клетки по поводу других состояний.

## Abstract (en)

Acromegaly is a neuroendocrine disorder caused by excessive production of growth hormone (GH). In the majority of cases the cause of acromegaly is a pituitary tumor producing GH. Cases of ectopic acromegaly are much rarer. Ectopic acromegaly occurs in cases of tumors which produce growth hormone-releasing hormone (GHRH) or extrapituitary tumors which produce GH. The main sources of excessive GHRH production are neuroendocrine tumors (NETs) of the lung or pancreas. Treatment of ectopic acromegaly consists of surgical removal of the source of GHRH hyperproduction and in cases where surgery is not an option, somatostatin analogues, pegvisomant, chemotherapy, immunotherapy or radiation therapy are used.In this article three cases of ectopic acromegaly due to GHRH-producing lung NETs are presented, each of them being notable for a number of features. In the first two cases, clinical symptoms were mild, besides in the second case ectopic acromegaly was accompanied by primary hyperparathyroidism. In the third case ectopic acromegaly was accompanied by pituitary macroadenoma, and after surgical removal of the lung NET remission of acromegaly was not achieved. In all three cases, lung NETs were detected incidentally on radiologic chest screening for other conditions.

## АКТУАЛЬНОСТЬ

Акромегалия — тяжелое хроническое нейроэндокринное заболевание, возникающее вследствие избыточной продукции гормона роста (соматотропного гормона, СТГ) [1]. Более чем в 95% случаев причиной акромегалии являются аденомы гипофиза, продуцирующие СТГ (как спорадические, так и семейные формы) [2][3]. Гораздо реже (менее 5%) встречаются случаи эктопической акромегалии [4]. Термин «эктопическая акромегалия», по сути, объединяет три понятия. Во-первых, это опухоли, продуцирующие гормон роста-рилизинг гормон (соматолиберин, ГРРГ), который, в свою очередь, стимулирует избыточную продукцию СТГ интактным гипофизом. К ним относятся как опухоли гипоталамуса (гамартомы, глиомы, ганглиоцитомы), при которых может развиваться гиперплазия, а затем СТГ-продуцирующая аденома гипофиза, так и нейроэндокринные опухоли (НЭО) или, редко, опухоли не нейроэндокринной природы различной локализации, продуцирующие ГРРГ [5]. Во-вторых, эктопические аденомы гипофиза, продуцирующие СТГ, располагающиеся за пределами турецкого седла — в клиновидной пазухе, пирамиде височной кости, полости носоглотки [6]. В-третьих, экстракраниальные опухоли, продуцирующие СТГ (например, НЭО поджелудочной железы [7], неходжкинская лимфома [8]). Первое описание эктопической акромегалии относится к 1949 г., когда Goldman описал пациента с сочетанием НЭО легкого, опухоли гипофиза и двусторонних образований надпочечников, предположив, что опухоль легкого вырабатывает фактор, стимулирующий рост опухолей в других органах [цитируется по 5]. Роль опухоли легкого как причины акромегалии была впервые доказана Altmann и Schütz, которые в 1959 г. сообщили о пациенте с клиническими проявлениями акромегалии, у которого не наступила ремиссия заболевания после облучения гипофиза, но заметно улучшилось состояние после резекции опухоли легкого [цитируется по 9]. В 1982 г. две независимые группы выделили белок, стимулирующий выработку СТГ (названный ГРРГ), из ткани опухоли поджелудочной железы у двух пациентов с акромегалией [10][11], что позволило установить причинно-следственную связь между развитием акромегалии и наличием опухоли внегипофизарной локализации.

К 2012 г. Garby и соавт. опубликовали обзор литературы, суммировавший данные о 53 известных случаях эктопической акромегалии [12], а Ghazy и соавт. — данные о 98 случаях [13]. В 2022 г. Zendran и соавт. опубликовали обзор, суммировавший данные о 127 известных случаях [14]. В целом в 2/3 случаях эктопическая акромегалия выявляется у женщин [13], а основными источниками секреции ГРРГ при эктопической акромегалии являются НЭО легких (типичные и атипичные карционоиды легких) (до 43%) и поджелудочной железы (до 35%) [14]. В единичных случаях могут встречаться феохромоцитомы, параганглиомы, лимфомы, тимомы и некоторые другие опухоли [12–14]. Только в двух случаях ГРРГ-продуцирующие опухоли имели не нейроэндокринную природу (диффузная крупная В-клеточная лимфома [15] и аденокистозная карцинома легкого [16]). Среди интракраниальных источников эктопической продукции ГРРГ чаще встречаются смешанные ганглиоцитомы-аденомы гипофиза [14].

Клиническая картина эктопической акромегалии не отличается от таковой при типичной акромегалии, а заподозрить ее можно при отсутствии визуализации аденомы гипофиза по данным магнитно-резонансной томографии (МРТ) головного мозга или при выявлении диффузной гиперплазии гипофиза [17]. Сложность диагностики эктопической акромегалии заключается в поиске источника продукции ГРРГ, а основным методом лечения является удаление первичной опухоли [13][14]. В случае невозможности радикального удаления опухоли могут быть назначены аналоги соматостатина длительного действия, пэгвисомант, химиотерапия, иммунотерапия, лучевая терапия [13][14].

В статье приведено описание трех клинических случаев эктопической акромегалии вследствие ГРРГ-продуцирующих НЭО легких, каждый из которых примечателен рядом особенностей. В России клинические случаи эктопической акромегалии ранее описаны не были.

## ОПИСАНИЕ СЛУЧАЕВ

## Случай 1

Пациентка С.Н.Ю. 1962 г.р., впервые госпитализирована в ГНЦ РФ ФГБУ «НМИЦ эндокринологии» Минздрава России в возрасте 58 лет. Считала себя больной в течение 6–7 лет, когда стала отмечать изменение внешности (отечность лица, увеличение размера стоп), снижение веса, общую слабость, раздражительность. При плановом осмотре гинекологом в 2014 г. заподозрена акромегалия, рекомендовано проведение МРТ головного мозга, в ходе которой визуализирована микроаденома гипофиза. В гормональном анализе крови выявлена гиперпролактинемия (исходные данные не предоставлены), была назначена терапия каберголином в дозе 0,25 мг один раз в неделю, на фоне чего достигнута нормализация уровня пролактина. В 2015 г. впервые определены повышенные концентрации инсулиноподобного фактора роста 1 (ИФР-1) и СТГ, установлен диагноз «Акромегалия». При контрольном обследовании по МРТ в 2016 г. сохранялась микроаденома гипофиза размерами 5х5 мм. С сентября 2016 г. в связи со стойкой нормопролактинемией каберголин отменен, начата медикаментозная терапия акромегалии аналогами соматостатина — октреотидом пролонгированного действия 20 мг 1 раз в 28 дней внутримышечно, который она получала в течение 6 месяцев. На фоне лечения пациентка отмечала улучшение самочувствия, однако ИФР-1 сохранялся повышенным. В связи с плохой переносимостью октреотида (уплотнение и гиперемия в местах инъекций) терапия была отменена. В декабре 2016 г. вновь выявлена гиперпролактинемия до 1098 мЕд/л, периодически возобновлялась и отменялась терапия каберголином. Динамики размеров аденомы гипофиза на фоне проводимого медикаментозного лечения аналогами соматостатина и каберголином отмечено не было.

В феврале 2017 г. во время плановой флюорографии у пациентки впервые выявлено объемное образование левого легкого. При проведении мультиспиральной компьютерной томографии (МСКТ) описано образование в корне левого легкого с четкими ровными контурами размерами 44х42х50 мм, неоднородной плотности с мелкими включениями кальция, плотностью 32–30 ед.Н. Образование было подозрительно в отношении злокачественного роста, поэтому в мае 2017 г. в онкологическом диспансере по месту жительства выполнена типичная сегментэктомия S4-5 слева, лимфаденэктомия. По данным иммуногистохимического (ИГХ) исследования удаленной опухоли, верифицирована высокодифференцированная НЭО легкого, Grade 1, индекс пролиферации Ki-67 менее 2%, что характерно для типичного карциноида. В послеоперационном периоде пациентка отметила значительное улучшение самочувствия: возвращение исходного веса (набор массы тела на 18 кг), исчезновение отеков лица, уменьшение общей слабости. При динамическом контроле гормональных показателей достигнута нормализация уровня ИФР-1 и гормона роста. После проведения МСКТ данных за рецидив опухоли не получено. У пациентки сохранялась гиперпролактинемия: уровень мономерного пролактина составил 1897 мЕд/л, микроаденома гипофиза размерами 4х5х5 мм, пациентка периодически получала терапию каберголином с положительным эффектом и продолжала наблюдаться у эндокринолога с диагнозом «Акромегалия», так как связи между удалением опухоли легкого и достижением ремиссии акромегалии установлено не было.

В 2020 г. у пациентки с акромегалией, микроаденомой гипофиза, НЭО легкого, повышением уровня хромогранина А до 169,12 мкг/л (0–100) был заподозрен синдром множественных эндокринных неоплазий 1 типа (МЭН-1), в связи чем она направлена на госпитализацию в ГНЦ РФ ФГБУ «НМИЦ эндокринологии» Минздрава России. В ходе стационарного обследования подтверждена ремиссия акромегалии: ИФР-1 — 177,2 нг/мл (17–238), минимальный уровень гормона роста в ходе перорального глюкозо-толерантного теста (ПГТТ) — 0,274 нг/мл. При физикальном обследовании отмечались мягкие проявления акромегалии (рис. 1). По данным МРТ головного мозга, на границе адено- и нейрогипофиза определялась слабо гиперинтенсивная на Т2 и гипоинтенсивная на Т1 ВИ структура, убедительно не накапливающая контрастный препарат, шириной 14,4 мм, высотой — до 7,3 мм, передне-задний размер — 2 мм. Образование наиболее соответствовало кисте кармана Ратке. В передне-правом отделе аденогипофиза по контуру дна и правого кавернозного синуса определялся участок слабо сниженного накопления контрастного препарата размерами 5х5,7 мм (микроаденома). Воронка не смещена. Отмечался диффузно сниженный МР-сигнал на Т2 ВИ (рис. 2). По данным дополнительного ИГХ-исследования, в удаленном карциноиде легкого экспрессии СТГ не обнаружено, выявлена диффузная экспрессия ГРРГ (рис. 3 А, Б).

**Figure:**
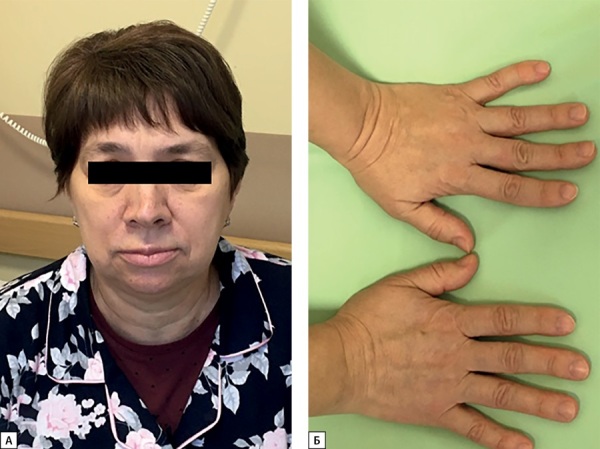
Рисунок 1. Пациентка С.Н.Ю. Типичные изменения внешности при мягкой форме акромегалии в пожилом возрасте: небольшое укрупнение черт лица (А), увеличение кистей (Б).

**Figure:**
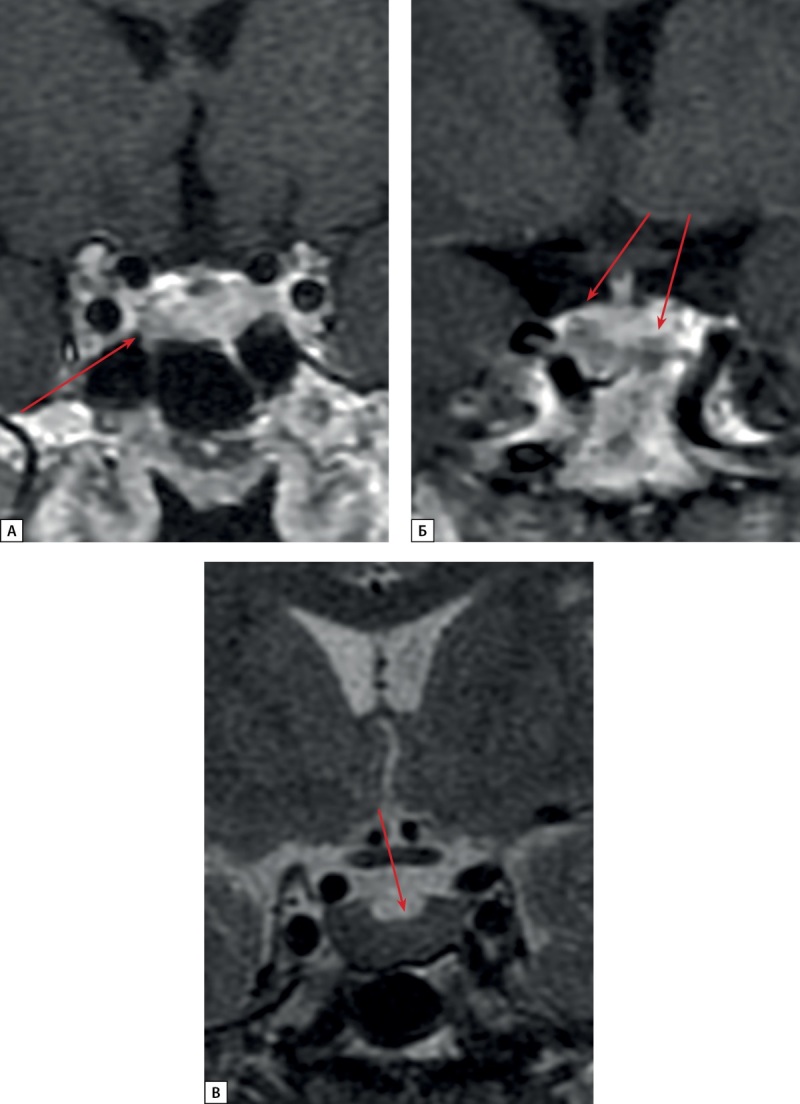
Рисунок 2. МРТ гипофиза пациентки С.Н.Ю., корональная проекция: А. Т1 ВИ с контрастном усилением. Микроаденома в левом отделе аденогипофиза (стрелка) Б. Т1 ВИ с контрастным усилением. Киста кармана Ратке (стрелки) В. Т2 ВИ. Диффузно-сниженный МР-сигнал на Т2 ВИ от ткани аденогипофиза (стрелка).

**Figure:**
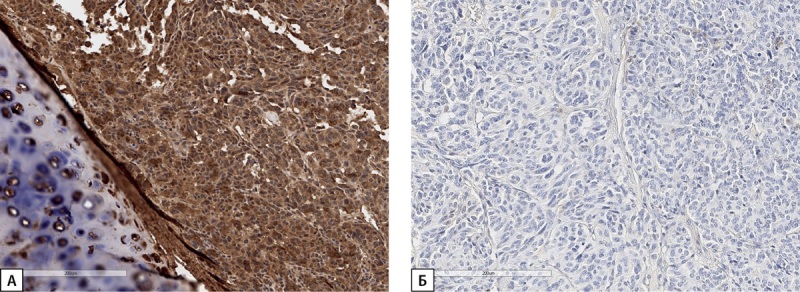
Рисунок 3. Иммуногистохимическое исследование карциноида легкого пациентки С.Н.Ю. (А) — диффузная экспрессия ГРРГ; (Б) — отсутствие экспрессии СТГ. Гистосканы.

В рамках обследования на предмет осложнений акромегалии данных за нарушение углеводного обмена не получено (глюкоза крови натощак — 4,88 ммоль/л, через 2 часа после нагрузки — 5,8 ммоль/л). Учитывая наличие многоузлового зоба в анамнезе, проведено ультразвуковое исследование щитовидной железы, в ходе которого выявлено увеличение объема до 27,7 мл с множественными узловыми образованиями размерами максимально до 3,8 см в диаметре в правой доле и до 1,3 см — в левой доле щитовидной железы. По данным тонкоигольной пункционной биопсии, подтвержден коллоидный в разной степени пролиферирующий зоб (Bethesda II). При гормональном обследовании — эутиреоз (ТТГ — 0,565 мМЕ/л, Т4св — 14,1 пмоль/л (9,0–19,0), Т3св — 3,5 пмоль/л (2,6–5,7), кальцитонин — 3,41 пг/мл (0–4,8). Осложнений со стороны сердечно-сосудистой системы у пациентки не обнаружено. В ходе колоноскопии исключены новообразования толстой кишки. В связи с жалобами на боли в спине выполнена рентгенография позвоночника, выявлен компрессионный перелом 12-го грудного позвонка, установлен диагноз: «Остеопороз смешанного генеза» и назначена антирезорбтивная терапия.

На фоне отмены каберголина и достижения ремиссии акромегалии обращало внимание сохранение повышения мономерного пролактина до 1116 мЕд/л (64–395). Для исключения возможных вторичных причин повышения уровня пролактина пациентка обследована на предмет гинекологических заболеваний — патологии не обнаружено. Из возможных причин гиперпролактинемии у пациентки имеется фиброзно-кистозная мастопатия. Учитывая умеренное повышение уровня пролактина, достижение нормопролактинемии на фоне приема небольшой дозы каберголина, отсутствие положительной динамики роста аденомы гипофиза, микроаденома гипофиза расценена как гормонально-неактивная. Учитывая сочетание микроаденомы гипофиза и НЭО легкого, проведено скрининговое обследование на предмет возможных компонентов синдрома МЭН-1: данных за первичный гиперпаратиреоз (ПГПТ), объемные образования поджелудочной железы, надпочечников не получено. Анализ крови на хромогранин А — 2,1 нмоль/л (менее 2).

В 2022 г. пациентка продолжила наблюдение в ГНЦ РФ ФГБУ «НМИЦ эндокринологии» Минздрава России: сохранялась стойкая ремиссия акромегалии, наблюдалась положительная динамика минеральной плотности костной ткани после введения золедроновой кислоты.

## Случай 2

Пациентка Д.И.Е. 1959 г.р., впервые госпитализирована в ГНЦ РФ ФГБУ «НМИЦ эндокринологии» Минздрава России в возрасте 60 лет с жалобами на выраженную общую слабость, головные боли, боли в суставах, потливость, снижение массы тела, отеки лица. Пациентка считала себя больной в течение последних двух лет, когда при обследовании после перенесенного гриппа было выявлено повышение глюкозы в крови до 9,3 ммоль/л, в последующем показатели гликемии нормализовались и была рекомендована диетотерапия. В связи с общим недомоганием пациентка обратилась к эндокринологу по месту жительства, и при обследовании было выявлено повышение уровня ИФР-1 до 349 нг/мл (норма до 200). По МРТ головного мозга отмечалась выраженная неоднородность структуры аденогипофиза слева, где нельзя было исключить аденому гипофиза. Центрально отмечалась гиперинтенсивная на Т2 ВИ структура размерами 3,8х1,5 мм, не накапливающая контрастный препарат (киста кармана Ратке). МР сигнал от аденогипофиза был нерезко снижен на Т2 ВИ (рис. 4 а, б).

**Figure:**
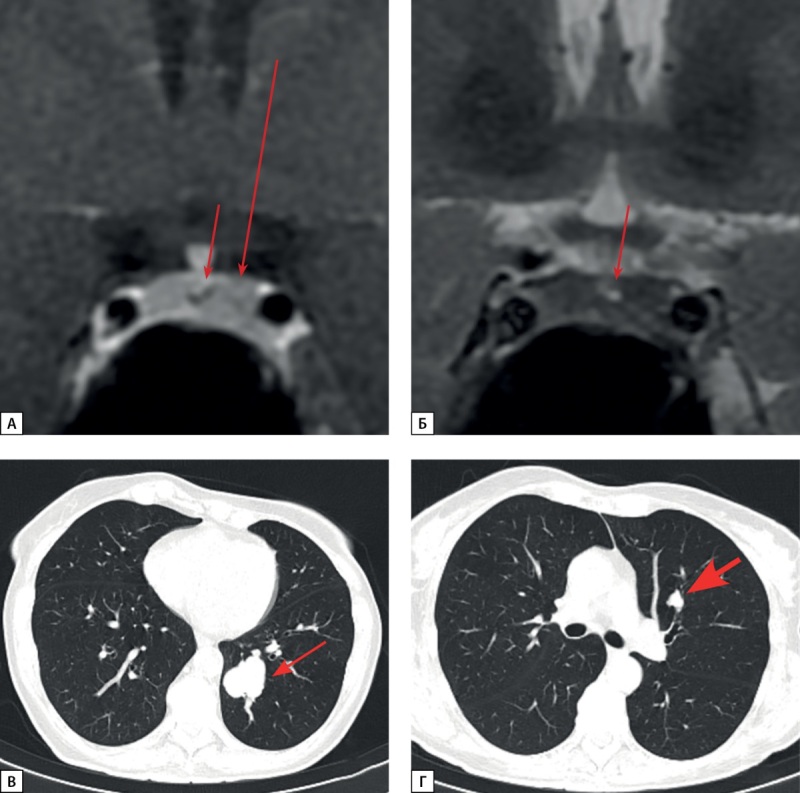
Рисунок 4. МРТ и КТ исследования пациентки Д.И.Е. а) МРТ гипофиза, корональная проекция, Т1 ВИ с контрастным усилением. Киста кармана Ратке (стрелка). Зона слабо сниженного накопления контрастного препарата в левом отделе аденогипофиза (длинная стрелка); б) Киста кармана Ратке (стрелка). Диффузно сниженный МР сигнал на Т2 ВИ от ткани аденогипофиза; в) МСКТ легких, легочный режим, нативная фаза, аксиальная проекция. Нейроэндокринная опухоль в S10 левого легкого (короткая стрелка); г) МСКТ легких, легочный режим, нативная фаза, аксиальная проекция. Образование в S3 левого легкого с внутри- и перибронхиальным распространением — очаг отсева (толстая стрелка).

При госпитализации в ГНЦ РФ ФГБУ «НМИЦ эндокринологии» Минздрава России подтверждена активная стадия акромегалии: ИФР-1 591,1 нг/мл (15–230), при проведении СТГ в ходе ПГТТ минимальное значение СТГ 2,53 нг/мл. Примечательно, что клинические признаки акромегалии были неярко выражены: небольшая отечность лица и укрупнение носа и пальцев рук. Также при обследовании впервые верифицирован диагноз ПГПТ: выявлено повышение уровня паратгормона до 78,8 пг/мл (15–65), кальций общий 2,51 ммоль/л (2,15–2,55), при ультразвуковом исследовании околощитовидных желез выявлено образование правой нижней околощитовидной железы 1,2х0,5х0,7 см. Из осложнений ПГПТ выявлена остеопения в 33% лучевой кости до -2,0 SD по Т-критерию. В феврале 2019 г. проведена трансназальная транссфеноидальная аденомэктомия. При проведении СТГ в ходе ПГТТ в раннем послеоперационном периоде максимальное подавление СТГ составило 0,89 нг/мл. После проведенного нейрохирургического вмешательства на гипофизе доставленный операционный материал был представлен тканью аденогипофиза с обширными полями оксифильных клеток, заподозрено наличие опухоли гипофиза. Однако на дополнительных срезах с импрегнацией серебром была обнаружена сохранная сеть ретикулиновых волокон с микрофокусами расширенных ацинусов, что соответствует гиперплазии аденогипофиза. При иммуногистохимическом исследовании большинство клеток были иммунопозитивные к СТГ (рис. 5).

**Figure:**
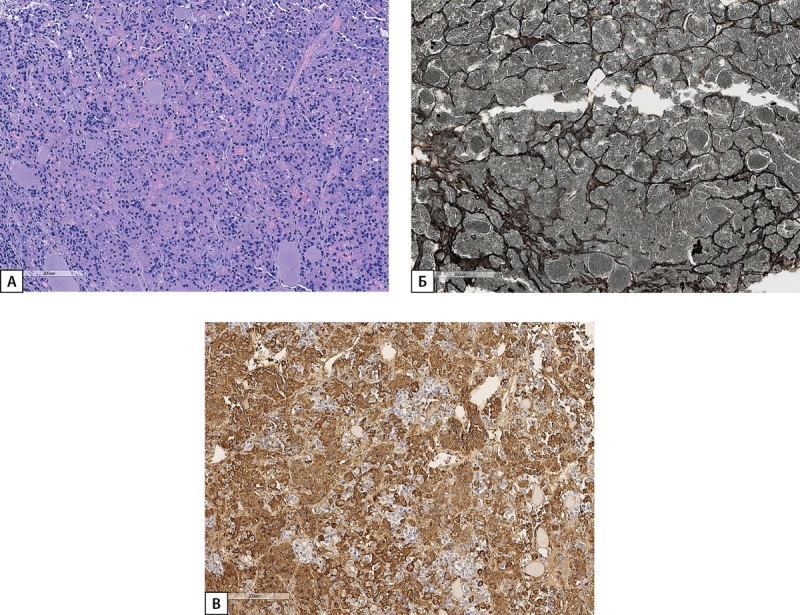
Рисунок 5. Гистологическое и иммуногистохимическое исследование операционного материала гипофиза пациентки Д.И.Е. (А) — ткань аденогипофиза с обширными полями оксифильных клеток (гемотоксилин-эозин). (Б) — сохранная сеть ретикулиновых волокон с расширенными ацинусами (импрегнация серебром). (В) — диффузное окрашивание клеток аденогипофиза СТГ (иммуногистохимическая реакция с антителами к СТГ). Гистосканы.

С учетом сочетания акромегалии и ПГПТ, с целью исключения синдрома МЭН-1, проведено высокопроизводительное параллельное секвенирование панели генов-кандидатов (AIP, CASR, CDKN1A, CDKN1B, CDKN1C, CDKN2A, CDKN2C, CDKN2D, DICER1, GNAS, CDC73, MEN1, POU1F1, PRKAR1A, PRKCA, PTTG2, SDHA, SDHB, SDHC, SDHD (общее покрытие кодирующих экзонов: 96,7%)), по результатам которого патологически значимых изменений выявлено не было.

При контроле ИФР-1 через 3 месяца после оперативного вмешательства сохранялось его повышение до 389,2 нг/мл (89–255). При повторной госпитализации в ГНЦ РФ ФГБУ «НМИЦ эндокринологии» Минздрава России через год после оперативного вмешательства подтверждено отсутствие ремиссии акромегалии: ИФР-1 414,5 нг/мл (17–238). Однако по МРТ головного мозга с контрастом убедительных данных за наличие остаточной ткани аденомы гипофиза получено не было. Была рекомендована терапия октреотидом пролонгированного действия, на фоне чего отмечалась нормализация уровня ИФР-1 до 196,6 нг/мл (93–224).

В возрасте 61 года пациентка перенесла новую коронавирусную инфекцию, и при проведении МСКТ органов грудной клетки в S10 левого легкого было выявлено объемное образование неправильной формы с четкими, полицикличными контурами размерами 32х32 мм, плотностью 34 ед.Н (рис. 4 в, г). Образование располагалось в просвете заднего базального бронха, а также перибронхиально в паренхиме S10. Определялась частичная консолидация паренхимы сегмента дистальнее образования (субсегментарные ателектазы). В S3 левого легкого определялось второе образование, с четкими неровными контурами, размерами 16х13 мм, плотностью 56 ед.Н. Образование также было расположено в просвете переднего бронха и в паренхиме S3. Регионарные лимфатические узлы не увеличены. По заключению: образование в S10 левого легкого наиболее соответствует НЭО. Субсегментарные ателектазы в S10 левого легкого дистальнее образования. Образование в S3 — очаг отсева. По месту жительства выполнена видеоторакоскопическая нижняя лобэктомия слева, лимфодиссекция слева. По результатам гистологического исследования: атипичный карциноид, Grade 2, максимальный размер опухоли 30 мм. В крае резекции бронха, краях резекции сосудов, висцеральной плевре, а также ни в одном из исследованных образцов опухолевый рост не обнаружен.

В связи с подозрением на эктопическую акромегалию, в ГНЦ РФ ФГБУ «НМИЦ эндокринологии» Минздрава России были пересмотрены гистологические препараты и проведено ИГХ-исследование удаленной опухоли. По заключению: в готовых гистологических препаратах, окрашенных гематоксилином и эозином, в ткани легкого обнаружена опухоль с признаками нейроэндокринной дифференцировки с 2 митозами, признаками инвазивного роста в бронх, 7 лимфоузлов с реактивными изменениями, без элементов опухолевого роста. В готовых гистологических препаратах, окрашенных с антителами к Ki-67, TTF-1, цитокератину АЕ1/3, CD56, обнаружена экспрессия TTF-1, цитокератина АЕ1/3, CD56 в клетках опухоли, индекс метки Ki-67=13,2%. При ИГХ исследовании с антителами к ГРРГ наблюдается его очаговая экспрессия в большинстве клеток опухоли умеренной или выраженной интенсивности, реакция к СТГ отрицательная (рис. 6).

**Figure:**
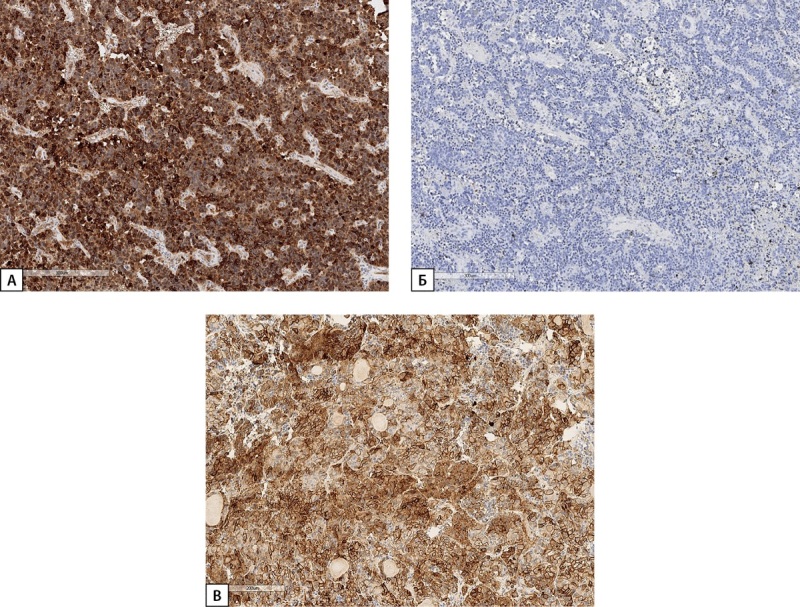
Рисунок 6. Иммуногистохимическое исследование карциноида легкого пациентки Д.И.Е. а) диффузная экспрессия ГРРГ; б) отсутствие экспрессии СТГ; в) положительная экспрессия рецепторов соматостатина 2А типа (SSTR 2A) (3 балла). Гистосканы

Таким образом, на основании результатов гистологического и ИГХ-исследования был установлен диагноз эктопической акромегалии. Было принято решение о пробной отмене аналогов соматостатина, на фоне чего показатель ИФР-1 оставался в пределах референсных значений (136 нг/мл (17–238)). При позитронно-эмиссионной КТ с 68Ga-DOTATAТЕ/DOTANOC, проведенной через 9 месяцев после оперативного вмешательства, признаков опухолевого роста не выявлено. Спустя 2,5 года после оперативного вмешательства у пациентки сохраняется ремиссия акромегалии. При контрольных обследованиях сохранялось повышение паратгормона до 102,5 пг/мл (15–65), при общем кальции 2,52 ммоль/л (2,15–2,55) и ионизированном кальции 1,33 ммоль/л (1,03–1,29), отсутствии вторичных причин повышения паратгормона. С учетом мягкого течения первичного гиперпаратиреоза (отсутствие остеопороза, мочекаменной болезни, отсутствие снижения скорости клубочковой фильтрации, нормокальциурия), выбрана тактика динамического наблюдения.

## Случай 3

Пациентка С.Н.А. 1978 г.р., впервые госпитализирована в ГНЦ РФ ФГБУ «НМИЦ эндокринологии» Минздрава России в возрасте 41 года с жалобами на головную боль, боли в спине, суставах, отечность лица, выпадение волос, увеличение веса, нарушения менструального цикла по типу задержек. Пациентка считала себя больной с возраста 37 лет, когда стала отмечать увеличение стоп и кистей, носа, онемение кончиков пальцев рук, задержки менструального цикла. В возрасте 40 лет обратилась к эндокринологу по месту жительства, при обследовании заподозрена акромегалия. Отмечено повышение уровня СТГ 8,29 нг/мл (0,5–7), однако данные уровня ИФР-1 на тот момент отсутствовали. По МРТ головного мозга с контрастным усилением выявлена макроаденома гипофиза с эндо-, супра-анте-, инфра-, латеро- (в левый кавернозный синус, KNOSP III) селлярным ростом, размерами 25х30х33 мм, компримирующая хиазму зрительных нервов (рис. 7а). Тогда же была выявлена железодефицитная анемия, ассоциированная с обильными менструациями на фоне миомы матки. По месту жительства в возрасте 41 года проведена трансназальная транссфеноидальная аденомэктомия. По результатам гистологического исследования — «крупноклеточная аденома гипофиза». После операции развился вторичный гипотиреоз, однако ремиссия акромегалии не была достигнута, и пациентка была направлена в ГНЦ РФ ФГБУ «НМИЦ эндокринологии» Минздрава России. Перед госпитализацией в связи с жалобами на хронический кашель пациентке была выполнена МСКТ органов грудной клетки, где было обнаружено образование верхней доли левого легкого 15х12 мм, расцененное онкологом как доброкачественное на основании КТ-фенотипа.

**Figure:**
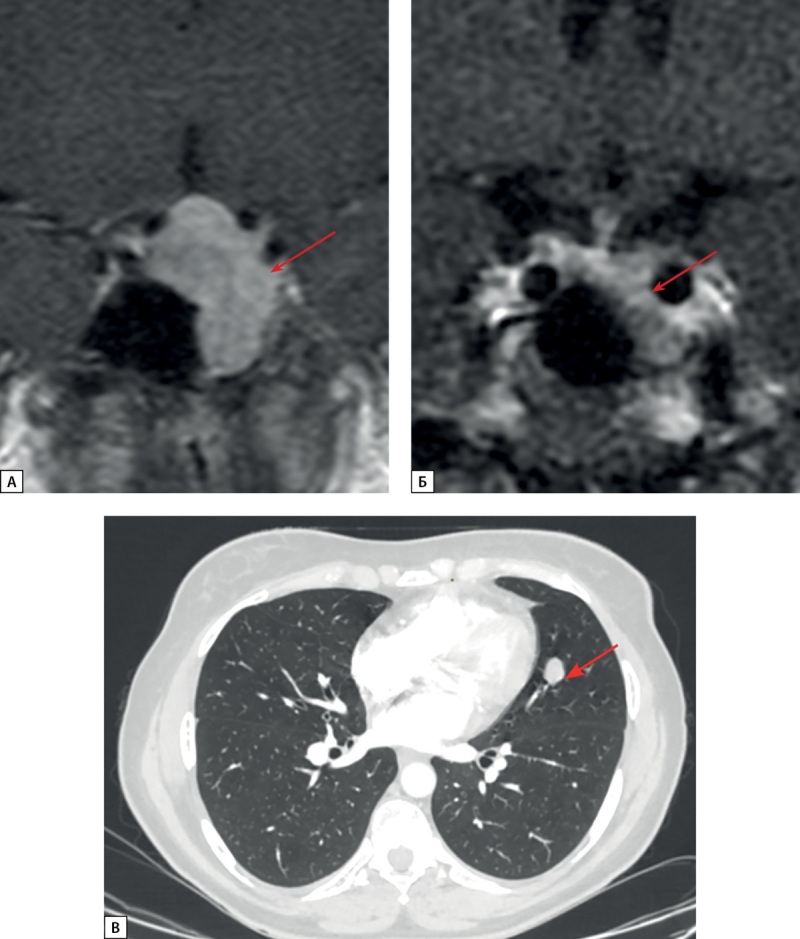
Рисунок 7. МРТ и КТ исследования пациентки С.Н.А. а) МРТ гипофиза до оперативного вмешательства, корональная проекция, Т1 ВИ с контрастным усилением. Макроаденома гипофиза (стрелка); б) МРТ гипофиза после оперативного вмешательства, корональная проекция, Т1 ВИ с контрастным усилением. Макроаденома гипофиза (стрелка), уменьшение размеров; в) МСКТ легких, легочный режим, артериальная фаза, аксиальная проекция. Нейроэндокринная опухоль в S4 левого легкого (стрелка).

При госпитализации в ГНЦ РФ ФГБУ «НМИЦ эндокринологии» Минздрава России во время обследования подтверждена активная стадия акромегалии: ИФР-1 538,9 нг/мл (64–395), отсутствовало подавление СТГ в ходе ПГТТ (минимальное значение — 1,03 нг/мл). По МРТ головного мозга с контрастным усилением в полости турецкого седла (слева), в клиновидных пазухах, больше слева, в левом кавернозном синусе (Knosp III) имеется кистозно-солидное объемное образование, неоднородно накапливающее контрастный препарат, размерами вертикальный — 25 мм, поперечный — 25 мм, переднезадний — 30 мм. При сравнении с МР-изображениями до проведения трансназальной транссфеноидальной аденомэктомии — умеренное уменьшение вертикального размера (расстояние до хиазмы 5 мм), распространение в латеральную часть клиновидной пазухи. По заключению: картина макроаденомы гипофиза с эндо-, инфра-, латеро- (S) селлярным ростом. Пациентка проконсультирована нейрохирургом, выполнена повторная трансназальная транссфеноидальная аденомэктомия. В операционном материале получена ткань опухоли гипофиза с иммунофенотипом плотногранулированной соматотропиномы (рис. 8). При проведении СТГ в ходе ПГТТ в раннем послеоперационном периоде отмечено подавление СТГ максимально до 0,6 нг/мл. В последующем по месту жительства в связи с отсутствием подавления СТГ в ходе ПГТТ был назначен октреотид пролонгированного действия в дозе 20 мг 1 раз в 28 дней внутримышечно. При повторном обследовании в ГНЦ РФ ФГБУ «НМИЦ эндокринологии» Минздрава России через год после операции, на фоне терапии октреотидом пролонгированного действия, сохранялось повышение ИФР-1 до 332,4 нг/мл (51–271). По МРТ головного мозга с контрастным усилением в полости турецкого седла, с распространением инфраселлярно в клиновидную пазуху, латероселлярно в левый кавернозный синус (KNOSP III), антеселлярно до клеток решетчатого лабиринта имеется кистозно-солидное образование неоднородной структуры, неравномерно накапливающее контрастный препарат, максимальными размерами: вертикальный — 18 мм, поперечный — 25 мм, переднезадний — 28 мм. Супраселлярная цистерна не сужена, хиазма зрительных нервов не компримирована. По сравнению с ранее выполненным исследованием — уменьшение объема опухоли (рис. 7б). С учетом отсутствия эффекта от двух проведенных оперативных вмешательств, пациентке было рекомендовано увеличение дозы октреотида пролонгированного действия до 30 мг 1 раз в 28 дней внутримышечно.

**Figure:**
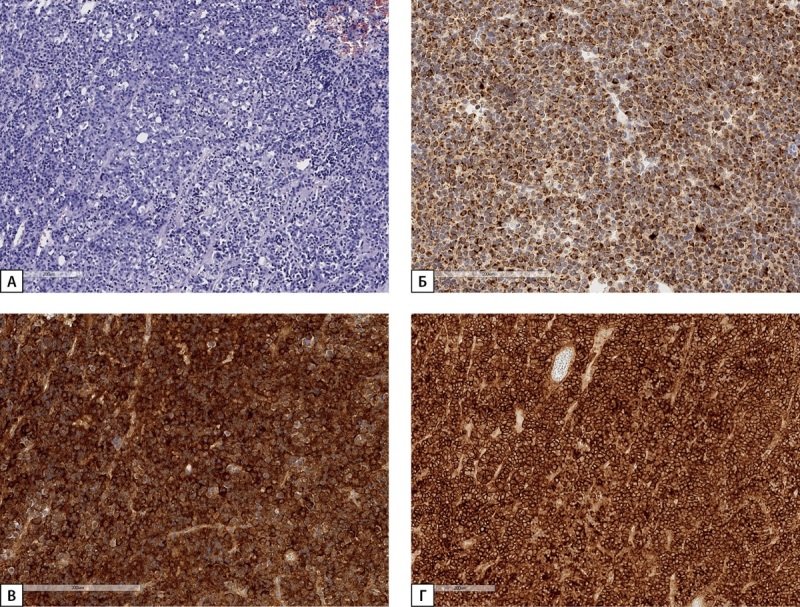
Рисунок 8. Гистологическое и иммуногистохимическое исследование операционного материала опухоли гипофиза пациентки С.Н.А. а) опухоль солидного строения из хромофобных клеток (гематоксилин-эозин); б) неравномерное цитоплазматическое окрашивание низкомолекулярным цитокератином, клон САМ 5.2 (иммуногистохимическая реакция); в) диффузная выраженная экспрессия СТГ в клетках опухоли (иммуногистохимическая реакция); г) выраженная экспрессия рецепторов соматостатина 2А типа 12 баллов по IRS (иммуногистохимическая реакция). Гистосканы.

В возрасте 44 лет пациентка была вновь госпитализирована в ГНЦ РФ ФГБУ «НМИЦ эндокринологии» Минздрава России для динамического наблюдения. На момент госпитализации получала октреотид пролонгированного действия в дозе 40 мг 1 раз в 28 дней (доза была увеличена по месту жительства в связи с отсутствием нормализации уровня ИФР-1). При обследовании подтверждено отсутствие медикаментозной ремиссии акромегалии: ИФР-1 300,8 нг/мл (51–271). По МРТ головного мозга с контрастом: в полости турецкого седла, в клиновидных пазухах (с распространением до задних отделов лабиринта), в левом кавернозном синусе сохраняется исходящее из аденогипофиза, неоднородно накапливающее контрастный препарат кистозно-солидное объемное образование неоднородной структуры, прежних размеров, размерами вертикальный — 18 мм, поперечный — 21 мм, переднезадний — 25 мм, без динамики по сравнению с предыдущим исследованием. Учитывая наличие в анамнезе объемного образования легкого, проведена МСКТ органов грудной клетки с контрастным усилением: в паренхиме S4 левого легкого периваскулярно визуализируется солидное образование с ровными несколько бугристыми контурами, размерами до 15х12х19 мм, умеренно накапливающее контрастный препарат (нативая/артериальная/венозная/отсроченная фазы исследования 13/15/69/79 ед.Н). По заключению: образование наиболее соответствует НЭО (рис. 7в). Проведена бисегментэктомия S4-5 слева с лимфодиссекцией 3, 5, 7, 9 групп. По результатам гистологического исследования: макроскопически на разрезах в толще ткани доли легкого определяется плотный, однородный, бледно-желтый опухолевый узел 1,6х1,5х1,3 см. Микроскопически опухолевый узел представлен плотноклеточным образованием из клеток со скудной слабо эозинофильной, нечетко контурированной цитоплазмой и мелкими овальными однотипными ядрами. Клетки формируют солидно-альвеолярные структуры, очаги некроза отсутствуют. Обнаруживается 3 митоза в 50 полях зрения при увеличении х400. Опухоль вовлекает стенку бронха, подрастает под эпителий его слизистой оболочки, суживает просвет бронха. Узел имеет четкую границу с окружающей легочной тканью, без сформированной псевдокапсулы. При ИГХ-исследовании в клетках опухоли диффузная экспрессия хромогранина А (клон DAK-A3, DAKO), Ki67 (клон SP6, Cell Marque) — ядерная экспрессия менее чем в 1% клеток. По заключению: типичный карциноид левого легкого, Grade 1, с поражением стенки субсегментарного бронха верхней доли, без элементов инвазии в краях резекции и без метастазов в 7 регионарных лимфатических узлах.

С учетом полученных результатов у пациентки была заподозрена эктопическая акромегалия. При проведении ИГХ-исследования выявлена диффузная экспрессия ГРРГ, что подтвердило диагноз эктопической акромегалии (рис. 9).

**Figure:**
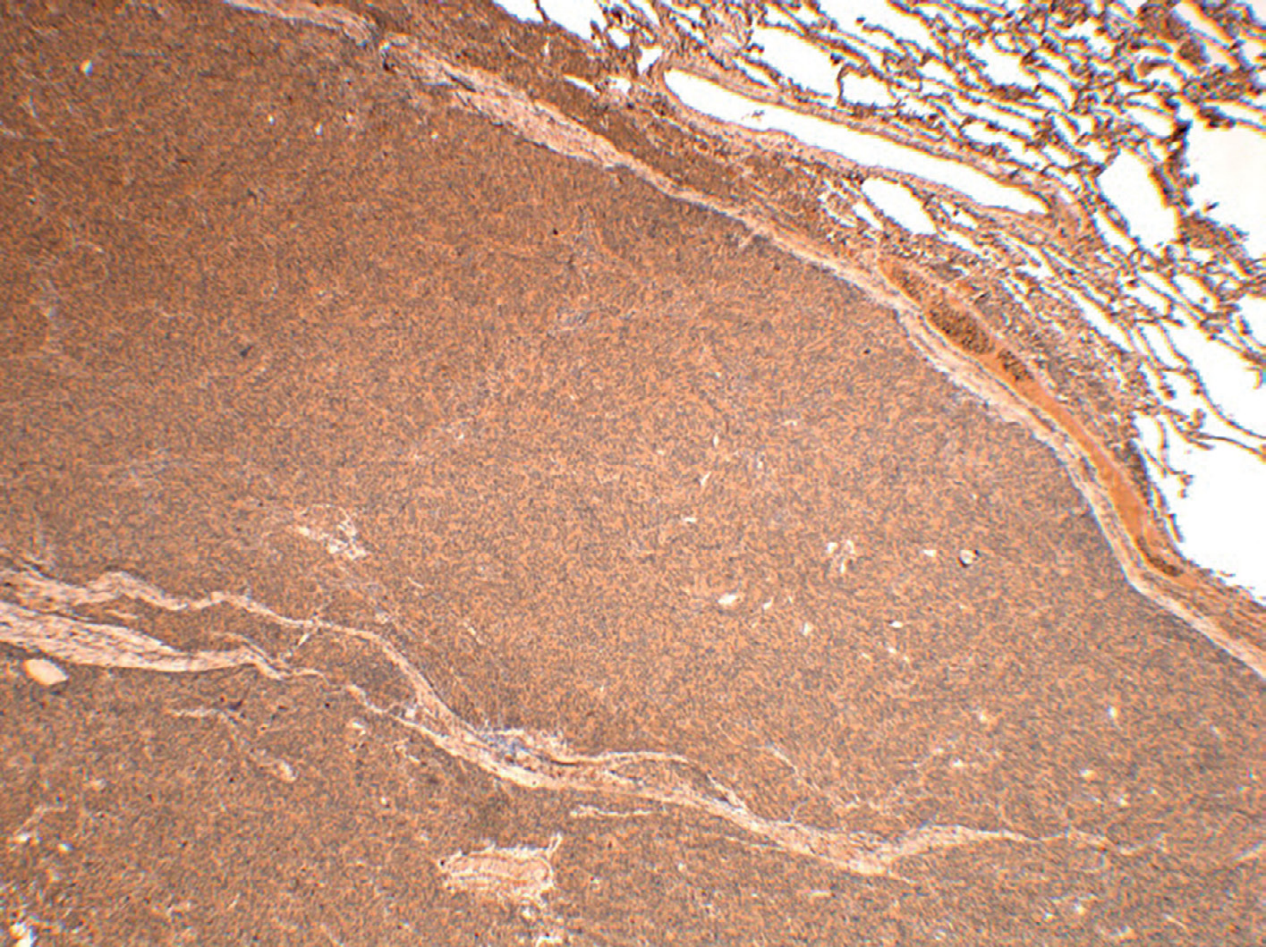
Рисунок 9. Иммуногистохимическое исследование карциноида легкого пациентки С.Н.А. Диффузная экспрессия ГРРГ. Ув 40.

Однако в послеоперационном периоде сохранялось повышение уровня ИФР-1, что потребовало продолжения терапии аналогами соматостатина. Сравнительные характеристики описанных случаев с эктопической акромегалией представлены в таблице 1.

**Table:** Таблица 1. Сравнительная характеристика пациентов с эктопической акромегалией

	Случай 1	Случай 2	Случай 3
Возраст на момент установления диагноза, лет	58	60	41
Пол	Женский	Женский	Женский
ИФР-1	Нет данных	591,1 нг/мл (15–230)	538,9 нг/мл (64–395)
СТГ базальный	Нет данных	Нет данных	8,29 нг/мл (0,5–7)
Максимальное подавление СТГ в ходе ПГТТ (менее 0,4 нг/мл)	Нет данных	2,53 нг/мл	1,03 нг/мл
Данные МРТ головного мозга	Новообразование гипофиза, соответствует микроаденоме гипофиза и кисте кармана Ратке	Выраженная неоднородность структуры аденогипофиза, подозрение на микроаденому гипофиза, киста кармана Ратке размерами 3,8х1,5 мм	Макроаденома гипофиза с эндо-, супра-, анте-, инфра-, латеро- (в левый кавернозный синус, KNOSP III) селлярным ростом, размерами 25х30х33 мм, компримирующая хиазму
Источник эктопии	Образование в корне левого легкого 44х42х50 мм. Гистологически: высокодифференцированная НЭО легкого, Grade 1, индекс пролиферации Ki-67 менее 2%, что характерно для типичного карциноида	Образование S10 левого легкого 32х32 мм, плотностью 34 ед.Н. В S3 левого легкого очаг отсева с четкими неровными контурами, размерами 16х13 мм, плотностью 56 ед.Н. Гистологически: атипичный карциноид, G2	Образование верхней доли левого легкого 15х12 мм. Гистологически: типичный карциноид левого легкого, Grade 1, с поражением стенки субсегментарного бронха верхней доли, без элементов инвазии в краях резекции и без метастазов в 7 регионарных лимфатических узлах

## ОБСУЖДЕНИЕ

В данной статье описаны три клинических случая эктопической акромегалии у женщин вследствие НЭО легких, что согласуется с данными литературы о том, что они являются самой частой локализацией НЭО, продуцирующих ГРРГ, и что чаще заболевание встречается у женщин [13][14]. Каждый случай примечателен рядом особенностей. В случае 1 отмечалось снижение веса, что нехарактерно для акромегалии, но может быть симптомом НЭО. Случай 2 характеризовался мягкими клиническими проявлениями акромегалии, хотя, как было указано выше, считается, что клинические проявления классической и эктопической акромегалии схожи [17]. У всех трех пациенток диагноз эктопической акромегалии был поставлен постфактум: в первом случае при ретроспективном анализе анамнеза пациентки, во втором и третьем — после ненаступления ремиссии акромегалии после трансназальной аденомэктомии и при случайном выявлении объемных образований легких по поводу других состояний.

Актуальным остается вопрос, на основании каких данных эндокринолог может заподозрить эктопическую продукцию ГРРГ у пациента с клинической картиной акромегалии. В 2022 г. Potorac и соавт. ретроспективно проанализировали данные МРТ головного мозга у 30 пациентов с эктопической акромегалией с целью выявления отличительных особенностей визуализации гипофиза в таких случаях [17]. По результатам у 24 пациентов была выявлена гиперплазия гипофиза, в двух случаях вертикальный размер гипофиза был несколько увеличен относительно нормы, в четырех случаях размер гипофиза был в норме. На основании анализа снимков МРТ авторы делают вывод, что гипофиз у пациентов с эктопической акромегалией слегка или умеренно увеличен в размерах, гипофиз гипоинтенсивен на Т2-взвешенных изображениях, при этом нормальная ткань гипофиза на МРТ не визуализируется. Кроме того, отсутствует инвазия в кавернозные синусы и компрессия хиазмы. Авторы выделяют следующие факторы, на основании которых можно заподозрить эктопическую акромегалию: гипоинтенсивное изображение гипофиза на Т2-взвешенных снимках (в 83,3% случаев), гиперплазия гипофиза (80%), средняя высота гипофиза — около 9,5 мм (редко — более 18 мм), нормальная ткань гипофиза не визуализируется, отсутствие инвазии в кавернозные синусы/клиновидную пазуху, отсутствие отклонения ножки гипофиза, отсутствие изменений в дне турецкого седла. Помимо МРТ-характеристик, авторы отмечают, что эктопическая акромегалия чаще встречается у женщин (73,3%), у мужчин заболевание проявляется в более молодом возрасте. Кроме того, в 50% случаев диагноз акромегалии был установлен раньше диагноза НЭО, самые частые локализации — легкие (56,7%) и поджелудочная железа (33,3%) [17].

Таким образом, отсутствие визуализации аденомы гипофиза у пациента с акромегалией, по данным МРТ, позволяет заподозрить эктопическую акромегалию. Тем не менее ряд работ указывают на то, что это не всегда так. В работе Lonser и соавт. среди 190 пациентов с акромегалией у 6 (3 мужчин и 3 женщин), по данным МРТ головного мозга, не визуализировалась аденома гипофиза, при этом трем пациентам была проведена МРТ по методике VIBE (volumetric interpolated breath-hold examination), что позволило обнаружить аденому гипофиза 4 мм в диаметре у одного из них. Всем пациентам была проведена эндоскопическая трансназальная ревизия гипофиза, позволившая обнаружить микроаденомы во всех случаях. Морфологическое и ИГХ-исследования подтвердили диагноз соматотропиномы во всех случаях, и у всех пациентов после операции была достигнута биохимическая ремиссия акромегалии [18]. Подобные наблюдения приведены и другими авторами [19].

В описанных нами случаях в первом имелась микроаденома гипофиза (наиболее вероятно, гормонально-неактивная), во втором случае — гиперплазия гипофиза, которая была исходно расценена как микроаденома гипофиза. Только в случае 3 имела место макроаденома гипофиза, что не типично для эктопической акромегалии. Возможно, макроаденома гипофиза сформировалась под длительным воздействием на гипофиз ГРРГ, и это послужило тому, что после хирургического удаления НЭО легкого не наступила ремиссия акромегалии. Случаев сочетания эктопической акромегалии с СТГ-продуцирующими макроаденомами гипофиза у одного пациента нами найдено не было.

Исследование уровня ГРРГ в плазме крови может иметь диагностическую значимость при подозрении на эктопическую акромегалию. Различными исследователями предлагаются следующие уровни концентрации ГРРГ в плазме, при которых можно ее заподозрить: более 300 нг/л [20] или более 250 нг/л [12]. В целом, существуют коммерческие наборы для определения ГРРГ, наиболее часто используемым из которых является ELISA [14]. Однако, учитывая редкость заболевания, небольшую доступность таких наборов, представляется целесообразным проводить топическую диагностику (исследование грудной и брюшной полости) при отсутствии визуализации аденомы гипофиза по МРТ головного мозга исходно, а также при отсутствии послеоперационной ремиссии акромегалии и отсутствии визуализации остаточной опухолевой ткани по МРТ головного мозга после операции.

В двух из трех описанных случаев (случаи 1 и 2) хирургическое лечение (удаление НЭО легких) привело к ремиссии акромегалии. В целом хирургическое лечение является лечением первой линии при эктопической акромегалии, поскольку позволяет полностью удалить источник гиперпродукции ГРРГ. При невозможности радикального удаления опухоли применяют различные другие виды лечения. Аналоги соматостатина длительного действия могут быть использованы в связи с наличием экспрессии соматостатиновых рецепторов 2 типа в НЭО [21]. При отсутствии экспрессии этих рецепторов в ткани опухоли аналоги соматостатина также могут давать частичный эффект в связи с воздействием на соматотрофы гипофиза, уменьшая выработку СТГ. В некоторых случаях прибегали к лучевой терапии на область гипофиза, а также химиотерапии, иммунотерапии, эмболизации метастазов, назначению пэгвисоманта [14].

Примечательно сочетание эктопической акромегалии и ПГПТ в случае 2. На основании сочетания акромегалии и ПГПТ у пациентки клинически был заподозрен синдром МЭН-1. В обзорной работе Zendran и соавт. указано, что в литературе описано 23 пациента с эктопической акромегалией и синдромом МЭН-1, при этом генетический анализ, подтверждающий диагноз, был проведен у 19 пациентов [14]. Примечательно, что в 18 случаях НЭО локализовалась в поджелудочной железе, в 1 случае — в тимусе, но не в легких. Помимо НЭО поджелудочной железы у большинства пациентов был ПГПТ [14], а у некоторых пациентов также имелись аденомы гипофиза (гонадотропинома [20], СТГ/пролактин-секретирующая аденома гипофиза [12], ноль-клеточная аденома [23]). Таким образом, у пациентов с МЭН-1 диагноз эктопической акромегалии вследствие ГРРГ-продуцирующей НЭО поджелудочной железы может быть затруднен ввиду возможного сочетания с аденомой гипофиза (гормонально-неактивной или с другим типом гормональной активности) в рамках этого синдрома. В целом, сочетание аденомы гипофиза и ПГПТ у одного пациента позволяет клинически верифицировать диагноз синдрома МЭН-1 [24]. При отсутствии выявления мутации в гене MEN1 данный случай может быть рассмотрен как фенокопия синдрома МЭН-1, и крайне редко у таких пациентов могут выявляться мутации в гене CDKN1B (синдром множественных эндокринных неоплазий 4 типа (МЭН-4)) [25]. У нашей пациентки (случай 2) мутации в генах MEN1 и CDKN1B не были выявлены. Учитывая отсутствие у нее отягощенного семейного анамнеза, а также тот факт, что при акромегалии повышена частота развития некоторых новообразований, возможно предположить либо что аденома околощитовидной железы развилась вследствие избыточного воздействия ИФР-1, либо случайное сочетание нескольких эндокринных опухолей у одного пациента.

По состоянию на 1 мая 2023 г. во Всероссийском регистре опухолей гипоталамо-гипофизарной области состоят 5726 пациентов с акромегалией. Исходя из эпидемиологических данных других стран, согласно которым 5% пациентов с акромегалией имеют внегипофизарную причину заболевания [4], более 250 человек, проживающих на территории Российской Федерации, должны иметь акромегалию, обусловленную эктопической секрецией СТГ или ГРРГ. Однако описаний таких пациентов в литературе российских авторов не найдено. К причинам несвоевременной (ретроспективной) диагностики эктопической акромегалии можено отнести низкую осведомленность врачей, в том числе эндокринологов, отсутствие специфических клинических проявлений, недоступность лабораторного измерения ГРРГ, высокую чувствительность НЭО к терапии аналогами соматостатина, отсутствие настороженности врачей в отношении пациентов с акромегалией, не достигших ремиссии заболевания после нейрохирургического лечения при радикальном удалении аденомы гипофиза.

## ЗАКЛЮЧЕНИЕ

Акромегалия встречается относительно редко в общей популяции, а эктопическая акромегалия в ее структуре — лишь в около 5% случаев. Тем не менее врачам-эндокринологам необходимо заподозрить наличие эктопической акромегалии при отсутствии визуализации аденомы гипофиза или при выявлении диффузной гиперплазии гипофиза, гипоинтенсивной на Т2-ВИ, по данным МРТ головного мозга. Диагностический поиск следует начинать с исключения НЭО легких и поджелудочной железы, а в случае выявления НЭО поджелудочной железы следует заподозрить синдром МЭН-1. Хирургическое лечение является методом выбора, поскольку потенциально позволяет полностью удалить источник гиперпродукции ГРРГ и привести к ремиссии акромегалии. В случае отсутствия ремиссии эктопической акромегалии после хирургического лечения целесообразно назначение различных вариантов медикаментозного лечения.

## ДОПОЛНИТЕЛЬНАЯ ИНФОРМАЦИЯ

Источники финансирования. Поисково-аналитическая работа и подготовка статьи проведены за счет гранта Российского научного фонда (проект №19-15-00398).

Конфликт интересов. Авторы декларируют отсутствие явных и потенциальных конфликтов интересов, связанных с содержанием настоящей статьи.

Участие авторов. Все авторы одобрили финальную версию статьи перед публикацией, выразили согласие нести ответственность за все аспекты работы, подразумевающую надлежащее изучение и решение вопросов, связанных с точностью или добросовестностью любой части работы.

Согласие пациента. Пациенты добровольно подписали информированное согласие на публикацию персональной медицинской информации в обезличенной форме в журнале «Проблемы эндокринологии».

## References

[cit1] Belaya Zhanna E., Golounina Olga O., Rozhinskaya Liudmila Y., Melnichenko Galina A., Isakov Michail А., Lutsenko Alexander S., Alekseeva Tatiana, Zenkova Tatiana S., Przhiyalkovskaya Elena G., Panyushkina Galina M., Ilukhina Olga B., Ivanova Elena I., Krishtal Ekaterina A., Vachygova Alla A., Pigarova Ekaterina A., Dzeranova Larisa K., Marova Evgenia I., Arapova Svetlana D., Mamedova Elizaveta O., Grebennikova Tatiana A., Antsiferov Mikhail B., Dreval Alexander V., Dedov Ivan I. (2020). Epidemiology, clinical manifestations and efficiency of different methods of treatment of acromegaly according to the United Russian Registry of Patients with Pituitary Tumors. Problems of Endocrinology.

[cit2] Sakhnova E. E., Przhiyalkovskaya E. G., Belaya Zh. E., Melnichenko G. A. (2022). Discordant parameters of insulin-like growth factor 1 and growth hormone in the diagnosis and monitoring of acromegaly. Problems of Endocrinology.

[cit3] Mamedova Elizaveta, Vasilyev Evgeny, Petrov Vasily, Buryakina Svetlana, Tiulpakov Anatoly, Belaya Zhanna (2021). Familial Acromegaly and Bilateral Asynchronous Pheochromocytomas in a Female Patient With a MAX Mutation: A Case Report. Frontiers in Endocrinology.

[cit4] Chanson P, Salenave S. Acromegaly. Orphanet J Rare Dis. 2008; 3:17. doi: https://doi.org/10.1186/1750-1172-3-17 PMC2459162 18578866

[cit5] SANO TOSHIAKI, ASA SYLVIA L., KOVACS KALMAN (2009). Growth Hormone-Releasing Hormone-Producing Tumors: Clinical, Biochemical, and Morphological Manifestations*. Endocrine Reviews.

[cit6] Ramírez Claudia, Hernández-Ramirez Laura-Cristina, Espinosa-de-los-Monteros Ana-Laura, Franco Juan Manuel, Guinto Gerardo, Mercado Moises (2013). Ectopic acromegaly due to a GH-secreting pituitary adenoma in the sphenoid sinus: a case report and review of the literature. BMC Research Notes.

[cit7] Melmed Shlomo, Ezrin Calvin, Kovacs Kalman, Goodman Robert S., Frohman Lawrence A. (2010). Acromegaly Due to Secretion of Growth Hormone by an Ectopic Pancreatic Islet-Cell Tumor. New England Journal of Medicine.

[cit8] Beuschlein Felix, Strasburger Christian J., Siegerstetter Volker, Moradpour Darius, Lichter Peter, Bidlingmaier Martin, Blum Hubert E., Reincke Martin (2002). Acromegaly Caused by Secretion of Growth Hormone by a Non-Hodgkin's Lymphoma. New England Journal of Medicine.

[cit9] Faglia G, Arosio M, Bazzoni N. Ectopic acromegaly. Endocrinol Metab Clin North Am. 1992; 21(3):575-595 1521513

[cit10] Guillemin Roger, Brazeau Paul, Bohlen Peter, Esch Frederick, Ling Nicholas, Wehrenberg William B. (2006). Growth Hormone-Releasing Factor from a Human Pancreatic Tumor That Caused Acromegaly. Science.

[cit11] Rivier Jean, Spiess Joachim, Thorner Michael, Vale Wylie (2004). Characterization of a growth hormone-releasing factor from a human pancreatic islet tumour. Nature.

[cit12] Garby Laetitia, Caron Philippe, Claustrat Francine, Chanson Philippe, Tabarin Antoine, Rohmer Vincent, Arnault Gwenaëlle, Bonnet Fabrice, Chabre Olivier, Christin-Maitre Sophie, du-Boullay Hélène, Murat Arnaud, Nakib Ihab, Sadoul Jean-Louis, Sassolas Geneviève, Claustrat Bruno, Raverot Gérald, Borson-Chazot Françoise (2012). Clinical Characteristics and Outcome of Acromegaly Induced by Ectopic Secretion of Growth Hormone-Releasing Hormone (GHRH): A French Nationwide Series of 21 Cases. The Journal of Clinical Endocrinology & Metabolism.

[cit13] Ghazi Ali A., Amirbaigloo Alireza, Dezfooli Azizollah Abbasi, Saadat Navid, Ghazi Siavash, Pourafkari Marina, Tirgari Farrokh, Dhall Dheepti, Bannykh Serguei, Melmed Shlomo, Cooper Odelia (2012). Ectopic acromegaly due to growth hormone releasing hormone. Endocrine.

[cit14] Zendran Iga, Gut Gabriela, Kałużny Marcin, Zawadzka Katarzyna, Bolanowski Marek (2022). Acromegaly Caused by Ectopic Growth Hormone Releasing Hormone Secretion: A Review. Frontiers in Endocrinology.

[cit15] Ravindra Vijay M., Raheja Amol, Corn Heather, Driscoll Meghan, Welt Corrine, Simmons Debra L., Couldwell William T. (2016). Primary pituitary diffuse large B-cell lymphoma with somatotroph hyperplasia and acromegaly: case report. Journal of Neurosurgery.

[cit16] Southgate H J, Archbold G P R, El-Sayed M E, Wright J, Marks V (2008). Ectopic release of GHRH and ACTH from an adenoid cystic carcinoma resulting in acromegaly and complicated by pituitary infarction. Postgraduate Medical Journal.

[cit17] Potorac Iulia, Bonneville Jean-François, Daly Adrian F, de Herder Wouter, Fainstein-Day Patricia, Chanson Philippe, Korbonits Marta, Cordido Fernando, Baranski Lamback Elisa, Abid Mohamed, Raverot Véronique, Raverot Gerald, Anda Apiñániz Emma, Caron Philippe, Du Boullay Helene, Bidlingmaier Martin, Bolanowski Marek, Laloi-Michelin Marie, Borson-Chazot Francoise, Chabre Olivier, Christin-Maitre Sophie, Briet Claire, Diaz-Soto Gonzalo, Bonneville Fabrice, Castinetti Frederic, Gadelha Mônica R, Oliveira Santana Nathalie, Stelmachowska-Banaś Maria, Gudbjartsson Tomas, Villar-Taibo Roció, Zornitzki Taiba, Tshibanda Luaba, Petrossians Patrick, Beckers Albert (2022). Pituitary MRI Features in Acromegaly Resulting From Ectopic GHRH Secretion From a Neuroendocrine Tumor: Analysis of 30 Cases. The Journal of Clinical Endocrinology & Metabolism.

[cit18] Lonser Russell R., Kindzelski Bogdan A., Mehta Gautam U., Jane John A., Oldfield Edward H. (2010). Acromegaly without Imaging Evidence of Pituitary Adenoma. The Journal of Clinical Endocrinology & Metabolism.

[cit19] Khadgawat Rajesh, Khandelwal Deepak, Mukund Amar, Suri Ashish (2011). Acromegaly with no pituitary adenoma and no evidence of ectopic source. Indian Journal of Endocrinology and Metabolism.

[cit20] Scheithauer Bernd W, Carpenter Paul C, Bloch Bertrand, Brazeau Paul (2004). Ectopic secretion of a growth hormone-releasing factor. The American Journal of Medicine.

[cit21] Gola Monica, Doga Mauro, Bonadonna Stefania, Mazziotti Gherardo, Vescovi Pier Paolo, Giustina Andrea (2006). Neuroendocrine tumors secreting growth hormone-releasing hormone: Pathophysiological and clinical aspects. Pituitary.

[cit22] Bertherat J, Turpin G, Rauch C, Li J Y, Epelbaum J, Sassolas G, Schaison G (2014). Presence of somatostatin receptors negatively coupled to adenylate cyclase in ectopic growth hormone-releasing hormone- and alpha-subunit-secreting tumors from acromegalic patients responsive to octreotide.. The Journal of Clinical Endocrinology & Metabolism.

[cit23] SHINTANI YASUMI, YOSHIMOTO KATSUHIKO, HORIE HIDEAKI, SANO TOSHIAKI, KANESAKI YOSHIKO, HOSOI EMIKO, YOKOGOSHI YUTAKA, BANDO HIROSHI, IWAHANA HIROYUKI, KANNUKI SEIJI, MATSUMOTO KEIZO, ITAKURA MITSUO, SAITO SHIRO (2008). Two Different Pituitary Adenomas in a Patient with Multiple Endocrine Neoplasia Type 1 Associated with Growth Hormone-Releasing Hormone-Producing Pancreatic Tumor: Clinical and Genetic Features.. Endocrine Journal.

[cit24] Thakker Rajesh V., Newey Paul J., Walls Gerard V., Bilezikian John, Dralle Henning, Ebeling Peter R., Melmed Shlomo, Sakurai Akihiro, Tonelli Francesco, Brandi Maria Luisa (2012). Clinical Practice Guidelines for Multiple Endocrine Neoplasia Type 1 (MEN1). The Journal of Clinical Endocrinology & Metabolism.

[cit25] Mamedova E.O., Mokrysheva N.G., Przhiyalkovskaya E.G. i soavt. Varianty i fenokopii sindroma mnozhestvennykh endokrinnykh neoplazii 1-go tipa. Terapevticheskii arkhiv. 2014; 86(10):87-91. 25509899

[cit26] Knyazeva O.V. Prediktory i chastota razvitiya novoobrazovanii shchitovidnoi zhelezy i zheludochno-kishechnogo trakta u patsientov s akromegaliei: Dis. ... kand. med. nauk. — Moskva; 2016 g. Dostupno po: https://www.endocrincentr.ru/sites/default/files/specialists/science/dissertation/1diss_kniazeva_v2.pdf?ysclid=lky09llvkf123399757 Ssylka aktivna na 10.08.2023 g.

